# A new pan-European Train-the-Trainer programme for bioinformatics: pilot results on feasibility, utility and sustainability of learning

**DOI:** 10.1093/bib/bbx112

**Published:** 2017-09-26

**Authors:** Allegra Via, Teresa K Attwood, Pedro L Fernandes, Sarah L Morgan, Maria Victoria Schneider, Patricia M Palagi, Gabriella Rustici, Rochelle E Tractenberg

**Affiliations:** 1 Istituto di Biologia e Patologia Molecolari Consiglio Nazionale delle Ricerche, c/o Dipartimento di Scienze Biochimiche "A. Rossi Fanelli", Sapienza Università, Roma, Lazio, Italy; 2 University of Manchester, School of Computer Science, Kilburn Building, Oxford Road, Manchester, United Kingdom of Great Britain and Northern Ireland; 3 Instituto Gulbenkian de Ciência, Apartado 14, OEIRAS, Portugal; 4 European Bioinformatics Institute, Cambridge, Cambridgeshire, United Kingdom of Great Britain and Northern Ireland; 5 University of Melbourne Melbourne Institute, Lab-14, 700 Swanston St, Melbourne Carlton, Victoria, Australia; 6 SIB Swiss Institute of Bioinformatics, CMU - 1 Michel Servet Geneva, Geneva, Switzerland; 7 University of Cambridge, Department of Genetics, Cambridge, Cambridgeshire, United Kingdom of Great Britain and Northern Ireland; 8 Georgetown University Medical Center, Neurology, suite 207 building D, 4000 reservoir rd., nw, washington, District of Columbia, United States

**Keywords:** Train-the-Trainers, pilot study, decision-making, degrees of freedom analysis, ELIXIR-GOBLET training

## Abstract

Demand for training life scientists in bioinformatics methods, tools and resources and computational approaches is urgent and growing. To meet this demand, new trainers must be prepared with effective teaching practices for delivering short hands-on training sessions—a specific type of education that is not typically part of professional preparation of life scientists in many countries. A new Train-the-Trainer (TtT) programme was created by adapting existing models, using input from experienced trainers and experts in bioinformatics, and from educational and cognitive sciences. This programme was piloted across Europe from May 2016 to January 2017. Preparation included drafting the training materials, organizing sessions to pilot them and studying this paradigm for its potential to support the development and delivery of future bioinformatics training by participants. Seven pilot TtT sessions were carried out, and this manuscript describes the results of the pilot year. Lessons learned include (i) support is required for logistics, so that new instructors can focus on their teaching; (ii) institutions must provide incentives to include training opportunities for those who want/need to become new or better instructors; (iii) formal evaluation of the TtT materials is now a priority; (iv) a strategy is needed to recruit, train and certify new instructor trainers (faculty); and (v) future evaluations must assess utility. Additionally, defining a flexible but rigorous and reliable process of TtT ‘certification’ may incentivize participants and will be considered in future.

## Introduction

Bioinformatics is a highly dynamic and rapidly evolving field that requires some level of understanding of the life sciences and of applied computation. This background must be augmented with an agility to respond to technological and computational advances, by using, adapting or developing new skills. Although there is a recognized global, unmet need for well-trained bioinformaticians across research domains [1–4], it is difficult to see how formal university programmes can meet this need effectively: the field changes so quickly that lengthy degree-level education may not be sufficient. University education is expensive in time and effort for students and instructors [4–5]; more importantly, it is too localized to meet the global demand. Clearly, it is neither possible nor desirable for all practising life scientists to complete new degrees; nevertheless, modern biological science requires at least awareness of, or familiarity with, basic bioinformatics terminology, tools and resources. These can include databases and algorithms such as those used in next-generation sequencing data analyses, resources collecting macromolecular data (e.g. UniProt) or genomic data (e.g. HapMap), graphical/visualization software (e.g. PyMol), databases of pharmaceutical interest (e.g. DrugBank) and alignment tools (e.g. BLAST).

To address the growing demand to build bioinformatics and computational abilities, ‘point-of-need’ training is becoming more prevalent, and is likely to remain relevant even if bioinformatics skills are incorporated into undergraduate curricula. Online opportunities have expanded dramatically, but (among many other challenges; [4]) the target skill set often requires both tutored hands-on practice and real-time feedback [6–7], making some online training (e.g. asynchronous and/or lecture-based ‘lessons’) less ideal. Locally available training in bioinformatics has therefore gained importance. However, experts in a field may themselves require training to competently teach/train and assess learners [8, 9]; in fact, instructors must be prepared with effective practices for delivering ‘point-of-need’, typically short-duration (e.g. half a day to 2 days), hands-on instruction. This is important because not only are ‘training’ and ‘education’ fundamentally different [10–11], but learning about teaching practices in either has not been a routine part of the professional preparation of life scientists in Europe. We use the term ‘best’ practices, meaning teaching practices based on long-standing ideas from cognitive science about how adults learn, e.g. [12–14] together with a consensus of experts in the domain and expert trainers ([15–17, 18]; see Table 5 for the definitions adopted in this article).

Training in ‘best’ practices for effective instruction is vital, but instruction and practise are also required to develop an understanding of the design, organization and evaluation of courses and teaching materials [5, 18]. To be truly ‘best’ practices, in both training and education, the delivered learning should be sustainable, i.e. should endure beyond the end of the instruction, and be applicable in other contexts [19, 20]. Experience with theory-based foundations is intended to help new instructors later to apply a general ‘best’ practice model [12, 13, 18] to their own future training courses; on its own, topic-specific training will not address the increasing demand for high-level skills that are responsive both to computational and technological innovations, and to their concomitant training [as suggested by 1–3, 4, 7]. Moreover, domain experts are embedded in their own universities or institutes, where training may not be their primary role. Offering training to those who do wish to become instructors can benefit their careers by exposing them to, and encouraging them to adopt, ‘best’ practices to improve the quality of their instruction [12–14].

Train-the-Trainer (TtT) is a model for establishing and growing a pool of individuals who are driven by their interest and enthusiasm to become (better) instructors. A notable large-scale, worldwide TtT programme that has established formal training and assessment protocols was developed by Software Carpentry (SWC) (http://software-carpentry.org) in 1998 and cloned for Data Carpentry (DC) (http://www.datacarpentry.org/), its sister organization, in 2014; the formal training model was first published in 2015 [4, 21]. The SWC and DC networks comprise volunteers who share the mission of delivering high-quality training in software development (SWC) or computational skills required for data management and analysis (DC). The TtT paradigms of both Carpentries follow a pedagogical theory-based structure (https://swcarpentry.github.io/instructor-training/), so that every trainer learns the same approach and method, specifically based on theory and best practices for training.

 Another training model was developed by the European Molecular Biology Laboratory-European Bioinformatics Institute (EMBL-EBI) (www.ebi.ac.uk/training/train-trainer), which has provided bioinformatics training and support to external and internal audiences since 2007, and has been running a TtT programme since 2012. Externally, the programme prepares new trainers to deliver a specific training course, which is based on an EMBL-EBI model [11]. Participants complete face-to-face training, attend and observe the course of interest and are then supported to develop, deliver and assess a version of the course that is appropriate to their target audience in their local context. Internally, the programme prepares new trainers to deliver the extant EMBL-EBI user training programme. After face-to-face training, internal (i.e. located at EMBL-EBI) trainers are assigned a mentor, and training opportunities are identified in which they can participate.

Each of these models has a specific focus: the SWC/DC TtT programme was designed to ensure consistent delivery of a consolidated set of training materials, developed as a community activity; the EMBL-EBI programme set out to enable external trainers to deliver courses in a similar manner to EMBL-EBI courses [11]. Both models have a network of trainers who have completed the TtT programme, which new instructors can access for expertise and support.

In 2015, ELIXIR (https://www.elixir-europe.org, https://f1000research.com/channels/elixir), in the context of the European Union H2020 programme (https://ec.europa.eu/programmes/horizon2020/), was granted additional funding, ELIXIR-EXCELERATE (https://www.elixir-europe.org/about-us/how-funded/eu-projects/excelerate) (EE), one of the objectives of which is to support a pan-European training programme to increase bioinformatics capacity and competency. This programme is not limited to the development of new courses and materials: it also specifically supports the development of new instructors to increase training capacity and sustainability, through a tailored TtT programme. The EE-TtT programme, its background and goals are described in detail in [22]. The formal preparation of new trainers via the EE-TtT aims to give new instructors tools and tips for providing an enriching learning experience to trainees, irrespective of topic, and to include best practice guidance on course and training material development.

The EE-TtT programme also aims to build a network of instructors to allow them to benefit from reciprocal support and discussion. The programme focuses on—but is not limited to—scientists in the ELIXIR community, aiming to promulgate ‘best’ practices and promote active learning ideas, grounded in educationally relevant, research-based theories of how people learn [12, 13, 18, 23].

A kick-off meeting was held in Hinxton, UK, in January 2016, bringing together ELIXIR training coordinators and global collaborators [including experts in cognitive and educational psychology, and representatives from SWC/DC and the Global Organisation for Bioinformatics Learning, Education and Training (GOBLET; [24])] to discuss the desired features of the new EE-TtT programme [22].

The programme was further developed during the following 4 months; four core topics were identified as the basis for the EE-TtT course structure: (1) principles of learning and how they apply to training; (2) training techniques for enhancing learner engagement and participation; (3) design of engaging sessions, materials and courses; and (4) assessment and feedback in training. These are described in detail in [22]. Seven pilot TtT sessions were then held from May 2016 to January 2017. This manuscript describes the evolution and features of this TtT programme, and feasibility and utility results from the pilot sessions. It also considers the sustainability of the learning TtT ‘graduates’ experienced, alongside lessons learned from the pilot project.

## Methods

### Pilot project structure

The EE-TtT pilot was created to identify and, through training, ‘qualify’ individuals to reliably and reproducibly train new users of bioinformatics and computational biology tools, methods and resources across Europe. The programme also aimed to seed a durable community that would support, nurture and mentor EE-TtT participants going forward. A pilot study was required to assess whether these goals could be accomplished satisfactorily; in the kick-off meeting, it was decided to call TtT course completers ‘new instructors’; those who were, or became, qualified to train new EE-TtT participants (i.e. able to lead the TtT workshops) were termed ‘TtT faculty’.

### TtT participants

Participants in the TtT kick-off meeting came from Europe and the United States. Fifteen participants were invited from the ELIXIR Training Coordinators Group (*n* = 10, representing Italy, The Netherlands, Portugal, Switzerland, UK and EMBL-EBI), from DC (1) and CyVerse (CyVerse (http://www.cyverse.org) mission is to design, deploy and expand a national cyberinfrastructure for life sciences research, and to train scientists in its use.) (1—by phone), and from the domain of educational-psychology-in-higher-education (4). Representation from GOBLET included its Chair, and most of the other participants were also members of GOBLET.

Participants in the TtT pilot sessions came from all over Europe, and were identified by their home ELIXIR node or through institutional affiliations with the ELIXIR nodes in their countries. Training sessions included 5–11 participants, and involved 2–3 TtT faculty (who had co-developed the materials in collaboration with GOBLET) with one exception, where the TtT lead faculty member was the sole instructor.

### Analyses

The evaluation of the pilot programme is a series of matrices based on the degrees of freedom analysis (DoFA) method [25, 26]. The DoFA method facilitates the analysis of qualitative data, including observations, survey results and theory. The method was applied to capture evidence from the pilot project about both the design and feasibility (or practicality) of the EE-TtT programme, and to study potential metrics for its utility (participant-perceived usefulness). Feasibility and utility were considered from the perspectives of the programme (ELIXIR and individual nodes) as well as faculty/participants. Finally, as the data were being collated and this report prepared, a new model of the sustainability (or potential for endurance and/or transfer of learning) was published [20]; hence, a final analysis examined the alignment of the EE-TtT programme with this model of sustainability of learning, as this is important for creating a programme that trains instructors who may then go on to become TtT programme developers themselves. The results are therefore organized to present the design of the programme (results of the kick-off meeting), the feasibility and utility of the materials and design, and then the sustainability of learning the TtT programme can promote. These aspects of the evaluation (feasibility; utility; sustainability of learning) are described in Table 5, together with descriptions of low, moderate and high levels of each of these features. The results of the evaluation are summarized in Table 5.

## Results

### Design

Table 1 is a DoFA matrix that shows the features of our two ‘model’ training programmes, as provided by SWC/DC and the EMBL-EBI. Features (rows) serve as predictions [26] on which we scored each of the models; scoring was derived from the kick-off meeting as follows: scores of 0 = model does not have this feature; scores of 0.5 = model has the feature, but it is either not sufficiently explicit or not as completely integrated as the experts involved in the EE-TtT programme design intended it to be; and scores of 1 = model has the feature and the EE-TtT programme can adopt that model’s implementation of it. Details about the execution of this DoFA analysis are given in Supplemental Table S1.

**Table 1:** DoFA: predictions of how/whether training models include the desired features of the EE-TtT programme

Features desired for the EE-TtT programme:	SWC/DC Carpentry TtT	EMBL-EBI TtT
Training paradigm is focused on theory and is evidence-based/evidence-informed	1	0.5
Explicit developmental trajectories—for the trainers themselves to continue to grow/refine their knowledge, skills and abilities (KSAs) relating to training specifically	0	0
Training paradigm seeks to build a community of ELIXIR trainers	0	0
Pedagogical and andragogical principles are explicit in training new trainers, so new instructors will also follow these principles	1	1
New trainers are introduced to—and encouraged to only use—evidence-based principles of learning	1	0.5
Training paradigm includes formative assessment of the training KSAs that the programme develops in new instructors	1	1
New trainers are introduced to—and encouraged to use—Bloom’s taxonomy to develop learning outcomes	1	1
Training paradigm embodies the target KSAs of effective training, course design and learning assessment, together with an explicit developmental trajectory new instructors can continue to build on	0	0
Training paradigm uses and promotes the use of active learning techniques	1	1
Programme provides instruction on how to integrate technology, including virtual machines (VMs) and cloud, in training delivery and development	0	0.5
Programme involves TtT participants attending actual training courses to observe expert instructors in action, and follow-up discussion about observation, evaluation and development of reflection around their own teaching	0.5	1
Programme includes post-TtT support (e.g. forum/blog/network/meetings/discussions), including support for instructors’ development of their own pre-course assessment (selection) and evaluations	1	1
Materials are FAIR	1	0.5

Table 1 shows that not all of the desired features of the EE-TtT programme were present in the two model training programmes, which supported the need for the pilot and the design of the EE-TtT programme itself. Although some features shown in Table 1 were adoptable or adaptable from the existing models, as noted earlier, these two TtT programmes were developed for specific purposes, and could not simply be adopted whole cloth. For example, the EE-TtT programme needs to build a network and community of trainers specifically around ELIXIR services and platforms. While both existing models are committed to the creation of communities for the trainers they prepare, those communities are not specific to ELIXIR services, platforms and resources. Another important feature for the new programme is that all training materials should be Findable, Accessible, Interoperable and Re-usable (FAIR). That is, the materials are intended to be made public (findable; accessible). This is true in one, but not the other, model. Finally, it is essential that the EE-TtT programme is based on theoretically driven ‘best’ practices in educational psychology, pedagogy and andragogy, to yield interoperable and reusable teaching practices that can be easily applied for new topics, new instructors and new tools/technology.

### Feasibility

Materials that implement all the desired characteristics outlined in Table 1 were developed to support 2 day training of new instructors [22]. These were used by the three faculty members who run the TtT programme to provide seven TtT sessions around Europe. Four key features of feasibility were identified. These are: (1) the financial costs to the organizers (hosts) of each TtT session; (2) costs to trainees who attend the session; (3) the number of participants; and the (4) feasibility of appending the TtT session to an existing conference, workshop, etc. (i.e. requiring an additional 2 day commitment to an already planned trip by organizers, faculty and trainees). Table 5 describes these features of ‘feasibility’ at high, moderate and low levels. Table 2 presents the feasibility results for each of the seven pilot sessions.

**Table 2:** **Feasibility results** ^a^

Feasibility factors:	Financial feasibility		Practical feasibility	
	Costs covered using EE-TtT budget	Costs that were not covered by the EE-TtT budget	# of participants (# of participants from the hosting node)	Stand-alone TtT course (1); co-organized with existing meeting (2) co-organized with other course (3)?
Case:				
Pilot 1 Cambridge—May 2016	Travel+hotel costs for one faculty	Coffee breaks and lunch; participants’ travel+hotel costs	11 (9)	1
Pilot 2 Cambridge—July 2016	Travel for one faculty	Coffee breaks and lunch	9 (9)	1
Pilot 3 Oeiras—July 2016	Travel+hotel costs for one faculty	Coffee breaks and lunch; trainees’ travel+hotel costs	8 (2)	3
Pilot 4 Rome—October 2016	Coffee breaks and lunch; travel+hotel costs for one faculty	Trainees’ travel+hotel costs	8 (0)	2
Pilot 5 Ljubljana—November 2016	Travel+hotel costs for one faculty	Coffee breaks and lunch One faculty+trainees travel+hotel costs	8 (4)	3
Pilot 6 Lausanne—January 2017	Coffee breaks; travel+hotel costs for one faculty	Trainees travel+hotel costs	9 (6)	3
Pilot 7 Oeiras—January 2017	None	Coffee breaks and lunch	10 (7)	1

^a^In general, coffee breaks, lunch and faculty travel costs were supported by the hosting node; they were not covered by the EE-TtT budget; Pilots 4, 6 and 7 were exceptions.

As for any event, the TtT programme requires resourcing to cover what can be considerable costs associated with both attending and hosting a TtT session. The full nature of these costs must be understood, and a model for recovering them devised, if the TtT programme is to be scalable (i.e. feasible going forward).

For scalability of the EE TtT programme, the pilot has uncovered a particularly important feature in terms of support for the interchange of EE-TtT faculty across sessions and countries. Scalability will only succeed with new faculty—especially in local nodes—and specifically, the provision of ongoing support (and possibly additional preparation) for those who have completed an EE-TtT workshop and wish to go on to deliver such workshops themselves. As can be seen in Table 2 and Table 5, the feasibility of a program must be understood from the perspectives of the funder (EE), the faculty and the attendees.

While experience with the pilot EE-TtT programme has shown that it can achieve its aim of ‘local capacity’ building, it has also underlined the need to develop a robust, sustainable cost model that would allow development, and incorporation, of new TtT faculty who can share the workload and allow unfettered roll-out of the programme across Europe. As the EE-TtT programme goes forward, all of these aspects need to be considered and evaluated. Feasibility going forward must be assessed from multiple perspectives (e.g. funder, faculty and participants; see Table 5) and on a variety of dimensions of the construct.

### Utility

We administered the same questionnaire (see Supplementary Materials) to participants at the end of every EE-TtT pilot but one to collect feedback. In Pilot 4, we administered a different questionnaire (see Supplementary Materials). The analysis of responses made it possible to assess the utility, based on user perspectives, of the EE-TtT programme.

The overall satisfaction was ‘good/excellent’ for every pilot (data not shown). However, to better understand whether participants perceived actual ‘utility’ from their engagement in this programme, we analysed the comments (given by <100% of participants in each pilot), to try to glean features of ‘perceived’ utility that could be incorporated into future workshop evaluations. We also included the yes/no responses to the question, ‘would you recommend the course?’ in this analysis as a ‘summary indicator’ of perceived utility. This item helps us to interpret whether what we sought as evidence of participant-perceived utility is plausible—based on the assumption that recommendations would not be made for a course that has no utility (as opposed to any utility). Table 3 therefore presents counts, by pilot session (rows), for how many of those who gave written additional feedback included a comment that is aligned with one or more of the themes (columns) we extracted from an informal analysis of the narrative responses.

**Table 3:** Perceptions of ‘utility’ gleaned from informal analysis of narrative comments in evaluations by participants across pilot sessions

Comment:	I learned new and relevant things about teaching and learning	I was inspired by new ways of thinking	Useful for excellence in future training that I offer/provide	Opportunities and a new venue to exchange ideas on teaching and learning with peers and TtT faculty	Useful practice and feedback on presentation skills	Useful practice and feedback on session/course preparation	Would you recommend the course? (Y: Yes N: No MB: Maybe)
Case^a^:							
Pilot 1 (*n*=9)		1^b^	2	3	4	1	Y: 8/9 N: 0 MB: 1/9
Pilot 2 (*n *=11)			2	4	5	5	Y: 10/11 N: 1/11 MB: 0
Pilot 3 (*n *=7)				4	2		Y: 5/7 N: 0 MB: 2/7
Pilot 4 (*n *=7)	–	–	–	–	–	–	Y: 5/7 N: 0 MB: 2/7
Pilot 5 (*n *=6)	1	6^d^	1		1	1	Y: 6/6 N: 0 MB: 0
Pilot 6 (*n *=6)	3		1	2	1		Y: 5/6 N: 0 MB: 1/6
Pilot 7 (*n *=9)	2	2	2	1	1	3	Y: 7 N: 1 MB: 1

^a^The number of individuals who did complete the course evaluation is given in parentheses.

^b^Table cells report the count of individuals who expressed a comment that is aligned with one or more of the themes (columns) we extracted from an informal analysis of the narrative responses to the feedback questionnaire we administered at the end of each TtT workshop (see Supplementary Materials).

^c^The version of this evaluation for Pilot 4 did not capture open-ended comments.

^d^The evaluation in this pilot workshop included the explicit question: ‘I was inspired to new ways of thinking’. In total, 6/6 people answered: ‘Agree completely’.

Considering Tables 2 and 3, we can compare and contrast ‘feasibility’ and ‘utility’. Specifically, the logistics for stand-alone TtT sessions are harder and more costly than for those that can be aligned with existing conferences or concurrent courses. ‘Feasibility’ is greatest when the TtT workshop is co-organized with another meeting that all participants (faculty and new instructors) have funding to attend, but the greatest ‘utility’ arises when the TtT workshop is co-organized with another course because participants have the opportunity to observe an experienced trainer teaching, which strengthens the training in many important ways. In this regard, of the 19 respondents to evaluations distributed in TtT workshops co-organized with another course (3/7 pilots), 10 provided an explicit positive comment on the importance of sitting in on a training session as observer/helper. This tension between utility and feasibility needs to be considered for the EE-TtT programme going forward.

Additionally, the features of perceived ‘utility’ that we extracted from this informal thematic analysis of responses to the open-ended course-evaluation questions must be considered for future evaluations: TtT evaluations should solicit actionable input about course utility and how to improve courses across all respondents. Further, the utility results in Table 3 are an informal analysis of what aspects of utility the participants perceived; collecting such valuable input should not depend on participants’ inclination to supply open-ended commentary in a field labelled ‘other comments’. Therefore, adding items to ask all participants to evaluate the utility of the TtT course is a priority for the scaled-up EE-TtT programme. Whether, or that, they have been inspired to think differently at the end of a course is important, but not sufficient, feedback: a further step is required to understand and evaluate true utility—specifically, we need to ascertain that they did something with the new knowledge and/or inspiration. Therefore, participant follow-up should also be put in place to ask whether they have done things differently in their post-course teaching and/or assessment.

### Sustainability

‘Sustainable learning’ is defined as learning that continues beyond the end of formal instruction, and can be described by four distinct features or dimensions that were originally identified in 2006 [19] and first demonstrated to be perceptible to students in 2017 [20]. The dimensions are:

1. Lifelong learning (an additional level of depth, or dimension, that you bring to a course or experience unrelated to the (primary) topic)
2. Changing your learning behaviour as a result of the specific learning: Describe how your learning (fact-finding, thinking, understanding of something or approach to learning something new) changed
3. A process of personal development continuing beyond the course: Something you did, or initiated, for your own sense of learning (i.e. not taking a course as part of your programme, but a learning or training experience that you sought, created or identified—not already planned)
4. Deconstruction/reconstruction: An idea or concept that you thought you understood, but recognized you did not truly understand (deconstruction), so sought deeper understanding, and discovered an error in your original understanding that you remedied or sought to remedy (reconstruction)

We used a DoFA to evaluate the alignment of the features of the EE-TtT programme with the four dimensions of sustainable learning—the alignment was achieved by the first and last authors’ independent evaluation, which was then discussed in a conference call to ensure consensus (disagreement was on two cells only). The final (consensus) version is explored in Table 4.

**Table 4:** Alignment of the EE-TtT programme with dimensions of sustainable learning

Sustainability dimensions:	Lifelong learning	Changing your learning behaviour as a result of the specific learning	A process of personal development continuing beyond the course	Deconstruction/reconstruction
TtT programme features:				
Training paradigm is focused on theory and is evidence-based/evidence-informed	X	X	X	X
Developmental trajectories—for the trainers themselves to continue to grow/refine their KSAs relating to training specifically	X	X	X	X
Training paradigm seeks to build a community of ELIXIR trainers	X	X	X	
Pedagogical and andragogical principles are explicit in training new trainers, so new instructors will also follow these principles		X	X	X
New trainers are introduced to—and encouraged to only use—evidence-based principles of learning		X	X	X
Training paradigm includes a formative assessment of the training KSAs that the programme develops in new instructors			X	
New trainers are introduced to—and encouraged to use—Bloom’s taxonomy to develop learning outcomes		X	X	
Training paradigm embodies the target KSAs of effective training, course design and learning assessment, together with an explicit developmental trajectory new instructors can continue to build on	X	X	X	X
Training paradigm uses and promotes the use of active learning techniques		X		X
Programme provides instruction on how to integrate technology, including VMs and Cloud, in training delivery and development				
Programme involves TtT participants attending actual training courses to observe expert instructors in action, and follow-up discussion about observation, evaluation and metacognitive development around their own teaching	X	X	X	X
Programme includes post-TtT support (e.g. forum/blog/network/meetings/discussions), including support for instructors’ development of their own pre-course assessment (for participant selection) and evaluations (for their continuing professional development)	X	X	X	
Materials are FAIR	X	X	X	X

The results in Table 4 suggest that the EE-TtT programme has potential to provide sustainable learning for the trainers who complete it. This is an important feature of a TtT programme because it can promote ongoing professional development, and lifelong learning around teaching and instruction—irrespective of what content or techniques need to be taught. The documentation of sustainability of the learning obtained via the EE-TtT programme would further support the focus on evidence and theory in the structure of the training that is provided. Adding items to the TtT course evaluation, or follow-up evaluations, that can assess the sustainability of the TtT learning would be useful for shaping this program towards this characteristic.

With this definition of sustainable learning in mind (see also Table 5), we can revisit the objective of the EE-TtT programme to create a durable community of supportive instructors and faculty. This objective would enhance the practicality of the programme because supporting new instructors and nurturing future TtT faculty make it more likely that the programme would become self-sustaining, as are the Carpentry communities. Establishing a community is only indirectly representative of ‘feasibility’, however. At this stage of the TtT programme’s development (immediately post-pilot), it is not possible for participants to perceive utility from such a feature—nor has there been opportunity yet to evaluate that perception. Nevertheless, the community aspect of the programme aligns with the sustainability of learning that participation in the TtT programme is intended to deliver. Although this was not considered when the pilot was being developed, sustainable learning is an important attribute that the programme can claim. As the programme scales up, attention to the cultivation of this community will be a priority.

**Table 5:** Key constructs for the TtT pilot program evaluation and summary of pilot results (in black ink) at ‘low’, ‘moderate’ and ‘high’ levels

Construct, definition	‘low’ levels	‘moderate’	‘high’
**Feasibility** (also practicality): From ELIXIR’s point of view: Is creating and maintaining (and growing) the program technically achievable and financially supportable?	Too costly Low interest/participation No faculty available to provide training Unacceptable levels of finances and time required	Breaks even/moderately costly Moderate interest/Participation Sufficient faculty <for now> Current funding and program are sufficient (no expansion is possible)	Affordable (or fully funded) Consistent, high interest and participation Faculty engaged and new faculty in training Evaluation and refinement of program can continue
**Feasibility** (also practicality): From TtT faculty point of view: Is participating as a faculty logistically achievable and financially reasonable?	Incentives to design/run new events low or negative Time commitment too great Disincentives to faculty participation from home institution	Moderate/positive incentives to design/run new events Time commitment acceptable No disincentives to faculty participation from home institution	Strongly positive incentives to design/run new events Time commitment acceptable. Incentives to faculty participation from home institution.
**Feasibility** (also practicality): From TtT participant’s point of view: Is participating logistically and financially reasonable?	• Too expensive to attend (time; money; effort); benefits of participation are not obvious	• Costs in time, money, and effort are balanced with benefits of participation.	• Costs in time, money and effort are far lower than benefits of participation
**Scalability:** From ELIXIR’s point of view: Can the program grow larger, effectively train new trainers to meet the growing demand for training—of new trainers and of new TtT faculty?	• Increasing program will greatly increase costs and requirements of time and expertise	• Increasing program will barely increase costs and requirements of time and expertise—but they will not go down	• Increasing program will decrease costs and requirements of time and expertise, as expertise is built in new trainees
**Utility:** Participant-perceived usefulness: Is completing this training beneficial or helpful to me at all?	• Low satisfaction with training; no interest in transferring new knowledge	Moderate satisfaction with training Some interest in transferring knowledge	• High satisfaction with training and concrete plans to actively transfer the knowledge
**Sustainability of the learning provided** [13,14]: Potential for endurance and/or transfer of learning from the formal setting to any other problem or setting	• No alignment with sustainable learning, limited chance for endurance or transfer of knowledge	• Some alignment with sustainable learning, moderate chance for endurance or transfer of knowledge	• Strong alignment with sustainable learning, significant chance to and even plans for both endurance and transfer of knowledge.
**‘Best’ practices:** Methods (of teaching) that are generally accepted to be superior to alternatives based on consistency (and level) of success	• ‘best’ is defined based on costs and not outcomes. Identified as ‘best’ because it is standardized	• ‘best’ is defined based on consensus of expert trainers. Identified as ‘best’ because it matches expert experiences	• ‘best’ is defined based on consensus of experts in the domain, expert trainers, and is aligned with strong evidence base. Identified as ‘best’ because it is evidence-based and also achievable

*Note*: Greyed out = not observed in the pilot results.

### Other lessons learned

Other results from the pilot study have significant implications for the future of the programme overall. These lessons relate to, or derive from, the definitions of scalability, feasibility and utility described in Table 5.

1. An important goal of the EE-TtT programme is to build training capacity in countries where the ability to provide bioinformatics training is not yet well developed. However, such countries may have difficulties in organizing or hosting a course; they may also be unable to cover the costs associated with sending newly qualified instructors to teach in other countries.
2. The ability to recruit trainees for EE-TtT sessions depends on the availability of funds (for the individual participants or nodes seeking to send participants) to support trainee participation. The EE-TtT pilot programme was necessarily constrained by grant-limited funds; however, the exercise highlighted features that must be factored into future cost models.
3. Another constraint on the ability to recruit trainees is that the priority they (or their home groups/institutions) give to training must often be balanced against competing commitments. Scientists need to be convinced that enhancing their training skills is likely to be useful to their careers.
4. A formal certification or recognition process is one possible strategy that could incentivize participation. This requires specific mechanisms via which trainees may be qualified, and maintain their qualifications. The training model used by the Carpentries requires that their trained instructors must teach two SWC/DC workshops every 2 years to maintain their certification. In our TtT kick-off meeting, we identified ongoing skills development, but not certification, as an important feature. However, as recognition is one of the principal themes that the joint ELIXIR-GOBLET training strategy (https://www.elixir-europe.org/news/elixir-and-goblet-publish-joint-training-strategy) sets out to address—and the EE-TtT programme already collaborates with GOBLET on material development—the natural next step will be to further collaborate on defining a process to promote trainer recognition and integrate this into the EE-TtT programme in future, if it is deemed a priority.
5. The EE-TtT pilot programme had only three ‘independent’ (confident to run a workshop with no assistance) TtT faculty members available, and highlighted the need to develop a structured programme to develop new faculty. As part of a trial process, one participant completed a TtT workshop, and then his home ELIXIR node hosted another workshop in which he served as an ‘assistant’, but expressed a sense of insufficient preparation to teach a workshop independently. Two other people attended a workshop (Pilot 6) with the aim of becoming TtT faculty, but the trial faculty development process stipulates that, in addition to attending the TtT workshop, participants must then also serve as ‘assistants’ or ‘co-trainers’ in at least another course. This model for preparing new EE-TtT faculty is therefore not robust enough to achieve the objective of developing—and retaining—new TtT faculty. We intend to explore the Carpentry model further for ways to refine EE-TtT faculty development.

Table 5 summarizes the findings of the evaluation of the pilot in terms of feasibility, utility, sustainability and scalability. The results of Tables 1–4 are summarized by integrating the evidence/lessons learned with the key constructs of the evaluation.

As can be seen, the results about feasibility depend on the perspective: there is some low, moderate and high feasibility from the ELIXIR point of view, only moderate feasibility for the participant’s perspective and both low and moderate feasibility from the faculty point of view. Similarly, scalability results are low and moderate. However, utility, sustainability and the alignment with ‘best’ practices are moderate (utility) to high.

## Discussion and conclusions

This pilot study has successfully identified strengths and weaknesses of the EE-TtT programme. Strengths include the intense commitment of faculty; alignment of the design with features of sustainable learning; evidence of participants’ perception of utility of the TtT workshops; and great deal of concrete actionable input as to next steps for further TtT workshop offerings and how best to evaluate them.

Weaknesses arise from the fact that the TtT faculty and the first cohorts of workshop participants were highly enthusiastic early adopters, so little information was gleaned from the wider community. For the TtT programme to be self-sustaining, and to encourage the development of a network and community around training new bioinformatics instructors, the identified challenges in feasibility/scalability must be addressed so that the program can be shown to work more broadly.

The pilot has highlighted several aspects on which to focus as the EE-TtT programme evolves, as detailed in the ‘Results’ section. We plan to complete a formal evaluation of the programme and its materials before formulating the next stage. Meanwhile, ancillary (positive) feedback has arisen from further piloting of the materials in two new TtT programmes, one for high school teachers and one for university faculty and doctoral students, currently being run by ELIXIR-Italy.

The EE-TtT programme was designed to have the essential features shown in Table 1; these differ sufficiently from other programmes to have justified the development of a new one. Moreover, Table 4 shows that the new programme, as developed, is aligned with features of sustainable learning. Sustainability of the learning that TtT delivers should be an evaluated feature of the scaled-up EE-TtT programme. To date, no TtT program has focused on or emphasized the sustainability of the learning that was delivered.

Some of the lessons learned from this pilot relate to the grant-limited nature of its current funding model: while stimulating local capacity building, it limited the mobility of experienced and new TtT faculty across Europe. This highlighted the need to develop a robust funding model to scale the TtT programme up successfully. Building a viable model of cost recovery for training is a challenge but is nevertheless essential. This pilot was initiated with grant funding, but both feasibility and scalability require ongoing investment. In this respect, we can learn from the SWC/DC and GOBLET approaches, and draw on the experience of dedicated training programmes, such as those provided by EMBL-EBI, EMBL-ABR, the Swiss Institute of Bioinformatics, the Gulbenkian Institute and the University of Cambridge, as examples. There have also been many virtual training platform efforts [27, 28] including a distance (‘e-’) learning platform being developed via ELIXIR at the Slovenian node [29]. The SWC/DC successfully delivers instructor training sessions online, allowing faculty to teach a large number of participants sitting in different countries, and overcoming scalability issues while also providing the hands-on, synchronous engagement that is so essential to successful learning of complex programming and software-based tools and methods. As these are features that make distributed training effective, the SWC/DC model is worth emulating to ensure that the EE-TtT follows suit (e.g. avoiding asynchronous and/or lecture-based programmes).

The pilot programme has also yielded actionable information about planning and hosting future TtT workshops, and has highlighted important choices about whether to prioritize utility or feasibility. Scheduling stand-alone TtT sessions is more costly (time/money), but was not perceived by participants to be more or less useful; feasibility was greatest when TtT sessions were scheduled alongside scientific meetings or conferences; but the greatest utility arose when the workshops were co-organized with other courses. Given this observation, utility would need to be explicitly assessed if e-learning approaches are used in future TtT offerings.

There were some challenges filling the TtT workshops. This is not really surprising, as most life scientists receive grant funding to support their research and not their professional development as instructors; it might therefore have been doubly difficult to justify taking time away from their research and funding their participation in TtT workshops. Moreover, while members of ELIXIR have an interest in increasing national training capacity, engagement in training and especially professional development are seldom the top priorities of life scientists. Hence, strategies may be required to convince researchers that TtT workshops can make significant contributions both to their work and to the scientific community more broadly.

One approach to incentivizing participation is to introduce a formal process of certification or qualification. The Carpentries’ training models (Table 1) could be a useful reference point; and although the committee ultimately decided that the approach would not be successful, GOBLET's work around certification and “badging” is also important to leverage, given the significant overlap of ELIXIR and GOBLET membership within the TtT programme (as faculty, participants and, thereby, future faculty). Whether a certification process is a priority will have to be determined in the programme’s next stages. Other possibilities include ‘fellowship’ awards and/or scholarship funding for which individuals can apply, which would add both the training and an ‘award’ to their CV. Options for incentivizing participation to be considered cannot create additional burdens (which could further disincentivize new participants).

Overall, these results show significant strengths and remediable weaknesses in the pilot EE-TtT programme, and we are committed to its further development. With the engagement of ELIXIR Training Coordinators, TtT faculty and contributors and GOBLET—which has as a core mission the ‘professionalisation’ of bioinformatics training—we will gain additional motivation, support and expert input in evaluating the EE-TtT programme, to help scale it up and drive it forward.

##

**Key Points**

- To increase bioinformatics training capacity across ELIXIR, a TtT programme has been created and piloted.
- Groundwork for the pilot involved designing a formal and theoretically based training paradigm that can support the preparation of future trainers to develop learning opportunities in the bioinformatics resources, tools and methods that European life scientists need.
- Feedback from seven pilot TtT sessions will support the ongoing success of the programme, including development of new faculty.
- Pilot results suggest that while the TtT programme is feasible, useful and consistent with principles of sustainable learning, issues of scalability remain to be addressed.
- Key outcomes have highlighted the need for: (i) support for organizational/hosting logistics, to allow instructors to focus their efforts on teaching; (ii) incentives for scientists to engage with opportunities to become new or better instructors; (iii) rigorous evaluation of the TtT materials to ensure they support sustainable learning; (iv) strategies to recruit, train and certify new instructor trainers; and (v) actionable items to be added to the evaluations to promote improvements to the programme overall.

## Funding

This work was funded by the European Union’s Horizon 2020 research and innovation programme ELIXIR – EXCELERATE, grant agreement no 676559.

## Supplementary Material

supplemental_materials_EE-TtT-feedback-questionnaire_bbx112

TTT_SupplementalTable_S2_dofa_bbx112

TTT_supplementalTable_S1_bbx112
