# Supplementary material for: A new pan-European Train-the-Trainer programme for bioinformatics: pilot results on feasibility, utility and sustainability of learning
Source: Brief Bioinform. 2017 Sep 26;20(2):405–15. doi: 10.1093/bib/bbx112 (PMC6433894; doi:10.1093/bib/bbx112)
Supplement: supplemental_materials_EE-TtT-feedback-questionnaire_bbx112 [file supplemental_materials_ee-ttt-feedback-questionnaire_bbx112.docx]

Supplemental Materials

**SM1. ELIXIR-EXCELERATE Train the Trainer Feedback Questionnaire**

Notes:

1. Q1/Q2 were optional

2. * required answer (select best response)

3. The version of this evaluation for Pilot 4 did not capture open-ended comments (i.e., without Q4A, Q9 and Q10).

4. Pilot 6 used a separate questionnaire (SM2).

Q1: Name (free text)

Q2: Email (free text)

Q3: Please tell us your overall rating for the entire course*

- Excellent
- Good
- Average
- Satisfactory
- Poor

Q4: Please rate each section of the course*

- Excellent
- Good
- Average
- Satisfactory
- Poor
- Did not attend

Q4A: Comments on Q4’s answer (free text)

Q5: What was the best part of the course? * (free text)

Q6: What was the worst part of the course? * (free text)

Q7: The balance of theoretical and practical content across the course was*

- Too practical
- About right
- Too theoretical

Q8: Would you recommend this course? *

- Yes
- No
- Maybe

Q9: Comments on Q8’s answer (free text)

Q10: Any other comments?

**SM2. Pilot 6 ELIXIR-EXCELERATE Train the Trainer Feedback Questionnaire**

**What is your area of research?**

**What is your career stage?**

**Gender**

F M Undecided

-------------------------------------------------------------------------------------------

**How do you rate the following course features?**

**Course organization**

Poor Average Good Very good Excellent

**Course announcement**

Poor Average Good Very good Excellent

**Registration process**

Poor Average Good Very good Excellent

**Course subject coverage (topics)**

Poor Average Good Very good Excellent

**Coverage of the proposed topics**

Poor Average Good Very good Excellent

**Total course duration**

Poor Average Good Very good Excellent

**Duration of the lectures**

Poor Average Good Very good Excellent

**Adequacy of the teaching methods**

Poor Average Good Very good Excellent

**Clarity of the lectures**

Poor Average Good Very good Excellent

**Balance between practicals and lectures**

Poor Average Good Very good Excellent

**Quality of handouts, references and other teaching aids**

Poor Average Good Very good Excellent

**Training room**

Poor Average Good Very good Excellent

**IT support**

Poor Average Good Very good Excellent

**ELIXIR-SI eLearning Platform (EeLP)**

Poor Average Good Very good Excellent

**Specific comments for EeLP?**

**Pace of teaching**

Poor Average Good Very good Excellent

**Social dinner**

Poor Average Good Very good Excellent

**Please use this place, if any of the questions needs further elaboration.**

------------------------------------------------------------------------------------

**How did you hear about this course?**

**Were there enough chances for you to discuss and network with the teaching staff and other participants?**

Yes No

**Would you recommend this course to other people?**

Yes No

**Please rate the overall quality of this course [0% to 100%]**

**Please rate how this course met your expectations [0% to 100%]**

**The topics were relevant for my work/research interests**

Disagree completely Disagree No strong feelings Agree Agree completely

**I was inspired to new ways of thinking**

Disagree completely Disagree No strong feelings Agree Agree completely

**What did you like most about the course?**

**What did you like least about the course?**

**Suggestions for improvements**

**Suggestions for future course themes**

Thank you very much for participating and for filling-in this feedback questionnaire!
