# Supplementary material for: A new pan-European Train-the-Trainer programme for bioinformatics: pilot results on feasibility, utility and sustainability of learning
Source: Brief Bioinform. 2017 Sep 26;20(2):405–15. doi: 10.1093/bib/bbx112 (PMC6433894; doi:10.1093/bib/bbx112)
Supplement: TTT_SupplementalTable_S2_dofa_bbx112 [file ttt_supplementaltable_s2_dofa_bbx112.docx]

Supplemental Table S2. Steps in Degrees of Freedom Analyses presented throughout the paper.

| **Step (adapted from Tractenberg, in press-2017)** | **Implementation (this paper)** |
| --- | --- |
| 1. Have/gain familiarity with the existing knowledge base about the phenomenon of interest sufficient to describe at least two options that represent the theory to be tested or decision to be made (columns). The prediction matrix is started here in step 1. | 1. Desired features of ideal training programme (**Table 1**) obtained from experts at kickoff meeting; Feasibility features were derived from the definition of “feasibility” in the context of the EE program (**Table 2**); Utility metrics were derived from the course evaluations from each pilot and their informal thematic analysis (**Table 3**); the model of sustainability of learning contains four key features (**Table 4**). |
| 2. Add rows to help make the decision from step 1. Identify at least one theory or framework that can inform or help justify, the decision; alternatively, add observations (data), as rows. This prediction matrix permits a visual and computational alignment of the decision (columns) with the evidence to be reviewed (rows; either theoretical features or data). | 2. Actual features of the two model training programmes (**Table 1**) were discerned by first and last co-authors; Feasibility data was obtained from the pilot sessions (**Table 2**); Utility data was obtained from the pilot sessions (**Table 3**); Desired features of ideal training programme were obtained from **Table 1** for exploration of their alignment with the four features of sustainability (**Table 4**). |
| 3. Evaluation of the alignment of the features of the chosen theory (or theories, in multiple matrices), with the decision options, is now possible. The rating system to be used (e.g., 0 for no alignment; 0.5 for some or possible alignment; and 1 for full alignment) should be determined prior to the evaluation (step 4). | 3. First and last co-author determined the rating system to be used (e.g., 0 for no alignment; 0.5 for some or possible alignment; and 1 for full alignment). The rating scales (point allocation) were revisited after step 4 (below) to ensure that results were interpretable. |
| 4. At least one independent judge evaluates each theory element or observation (rows) with respect to the decision options (columns), according to the a priori rating scale. It is helpful to consult an expert on education theory (e.g., institutional resource or colleague); otherwise, consensus among those involved in the decisionmaking itself (at least two) is advisable. Including explanation of the rating in each cell can be helpful to explicate the choice of ratings. | 4. First and last co-author rated each intersection of feature and data (i.e., each cell of each matrix). Ratings were generated independently by these two evaluators, discussed to reach consensus when agreement was not 100% initially (one row in Table 4); and then reviewed and discussed with co-authors. |
| 5. The “degrees of freedom” can be computed by summing the “points” in each column, the marginals. Column marginals help identify the theory (column) with the highest total evidence support, the “best supported theory”. Row mariginals can be useful to identify the most “theoretically consistent” observations, if that is of interest. If useful, a chi square statistic can be computed and its p-value estimated. | 5. The “degrees of freedom” are computed as the column marginals; however, simple visualization was all that was needed in these analyses. |
