# Supplementary material for: A new pan-European Train-the-Trainer programme for bioinformatics: pilot results on feasibility, utility and sustainability of learning
Source: Brief Bioinform. 2017 Sep 26;20(2):405–15. doi: 10.1093/bib/bbx112 (PMC6433894; doi:10.1093/bib/bbx112)
Supplement: TTT_supplementalTable_S1_bbx112 [file ttt_supplementaltable_s1_bbx112.docx]

Supplemental Table S1. Key constructs, their definitions, and how each looks when present at “low”, “moderate”, and “high” levels.

| **Construct, definition** | **“low” levels look like…** | **“moderate” levels look like…** | **“high” levels look like** |
| --- | --- | --- | --- |
| **Feasibility**  (also, practicality): From ELIXIR’s point of view: is creating and maintaining (and growing) the program technically achievable and financially supportable? | - Too costly - Low interest/   participation   - No faculty available to provide training - Unacceptable levels of finances and time required | - Breaks even/ moderately costly - Moderate interest/ - participation - Sufficient faculty <for now> - Current funding and program are sufficient (no expansion is possible) | - Affordable (or fully funded) - Consistent, high interest and participation - Faculty engaged and new faculty in training - Evaluation and refinement of program can continue |
| **Feasibility**  (also, practicality): From TtT faculty point of view: is participating as a faculty logistically achievable and financially reasonable? | - Incentives to design/run new events low or negative. - Time commitment too great. - Disincentives to faculty participation from home institution. | - Moderate/ positive incentives to design/run new events. - Time commitment acceptable. - No disincentives to faculty participation from home institution. | - Strongly positive incentives to design/run new events. - Time commitment acceptable. - Incentives to faculty participation from home institution. |
| **Feasibility**  (also, practicality): From TtT participant’s point of view: is participating logistically and financially reasonable? | - Too expensive to attend (time, money, effort); benefits of participation are not obvious. | - Costs in time, money, and effort are balanced with benefits of participation. | - Costs in time, money, and effort are far lower than benefits of participation. |
| **Scalability** (from ELIXIR’s point of view): can the program grow larger, effectively train new trainers to meet the growing demand for training – of new trainers and new TtT faculty? | - Increasing program will greatly increase costs and requirements of time and expertise. | - Increasing program will barely increase costs and requirements of time and expertise – but they won’t go down. | - Increasing program will decrease costs and requirements of time and expertise as expertise is built in new trainees. |
| **Utility**: participant-perceived usefulness: is completing this training beneficial or helpful to me at all? | - Low satisfaction with training; no interest in transferring new knowledge. | - Moderate satisfaction with training; some interest in transferring knowledge. | - High satisfaction with training and concrete plans to actively transfer the knowledge. |
| **Sustainability** **of the learning provided** [13,14]: potential for endurance and/or transfer of learning from the formal setting to any other problem or setting. | - No alignment with sustainable learning, limited chance for endurance or transfer of knowledge. | - Some alignment with sustainable learning, moderate chance for endurance or transfer of knowledge. | - Strong alignment with sustainable learning, significant chance to, and even plans for, both endurance and transfer of knowledge. |
| “**Best” Practices**: Methods (of teaching) that are generally accepted to be superior to alternatives based on consistency (and level) of success. | - “best” is defined based on costs and not outcomes. Identified as “best” because it is standardized. | - “best” is defined based on consensus of expert trainers. Identified as “best” because it matches expert experiences. | - “best” is defined based on consensus of experts in the domain, expert trainers, and is aligned with strong evidence base. Identified as “best” because it is evidence based and also achievable. |
